# Extended Analysis of Status Epilepticus and Cluster Seizures in Dogs in the Context of Overall Epilepsy Incidence: 254 Cases

**DOI:** 10.3390/ani15192807

**Published:** 2025-09-26

**Authors:** Martinas Jankauskas, Aistė Gradeckienė, Sigitas Čižinauskas, Olli Saalasti, Dmitrij Kvitka, Vita Riškevičienė

**Affiliations:** 1 Veterinary Academy, Lithuanian University of Health Sciences, LT-47181 Kaunas, Lithuania; aiste.gradeckiene@lsmu.lt (A.G.); dmitrij.kvitka@lsmu.lt (D.K.); vita.riskeviciene@lsmu.lt (V.R.); 2 Referral Animal Clinic Aisti, 01600 Vantaa, Finland; sigitas.cizinauskas@aisti.info (S.Č.); olli.saalasti@aisti.info (O.S.)

**Keywords:** status epilepticus, cluster seizures, seizure onset, canine epilepsy

## Abstract

Epilepsy is one of the most common chronic neurological disorders in dogs, characterized by recurrent, unprovoked seizures. Epileptic seizures are typically classified as single generalized (SG), cluster seizures (CS), or status epilepticus (SE), with CS and SE representing neurological emergencies linked to high morbidity and mortality. This retrospective study analyzed 254 canine epilepsy cases from two veterinary clinics in Finland and Lithuania, aiming to evaluate the frequency and clinical associations of CS and SE. Patient data included age of seizure onset, sex, weight, neuter status, blood test results, clinical and neurological examinations, magnetic resonance imaging (MRI) findings, etiology classification and clinical outcome. Seizure type distribution was as follows: CS in 47.2%, SG in 26.8%, and SE in 26.0% of cases. SG seizures were significantly more common than CS in dogs aged 6–72 months (54.4% vs. 38.3%, *p* = 0.005), and more frequent in first-time cases compared to previously treated ones (*p* = 0.005). SE was significantly associated with inflammatory brain disease, abnormal clinical and neurological findings, and a higher rate of euthanasia (29.2%, *p* < 0.05). Dogs with structural epilepsy were older (median 108 months) and more likely to develop SE (35.5%). Most cases initially presented as SG (58.1%), but 44.5% progressed to CS and 24.6% to SE. MRI revealed lesions in 46.5% of cases, with SE more frequent in dogs with inflammatory changes and SG predominating in neoplastic cases. No significant associations were found between seizure type and sex, reproductive status, or body weight. These findings provide new insights into seizure presentation, progression, and underlying pathology, supporting the importance of early diagnosis and individualized clinical management in canine epilepsy.

## Simple Summary

Epilepsy is one of the most common brain disorders in dogs. In some cases, seizures occur in dangerous patterns: many seizures in a short period (cluster seizures), or very long seizures (status epilepticus). These conditions are medical emergencies and can lead to poor outcomes. This study’s scope were 254 dogs diagnosed with epilepsy from two veterinary clinics in Finland and Lithuania. We evaluated the dogs’ age, sex, type of seizures, blood, clinical, neurological, MRI test results, and treatment outcomes. The findings show that cluster seizures were the most common, and dogs with status epilepticus had the worst outcomes, often needing euthanasia. Older dogs were more likely to have serious seizure types and brain problems seen on MRI. This study helps veterinarians better understand how seizure types relate to causes and outcomes and may help guide prognostic factors in the future.

## 1. Introduction

Epilepsy is rated as the most common neurological disease in both humans and dogs [1,2,3]. Hypersynchronous neuronal electrical activity in the cerebral cortex that manifests as a paroxysmal and transient abnormality of consciousness, motor activity, autonomic function, sensation, or cognition is called an epileptic seizure [3]. In veterinary medicine, the prevalence of patients experiencing epileptic seizures varies from 0.5% to 5.7% in the population [1,2,3,4,5,6,7,8,9,10,11].

According to the International Veterinary Epilepsy Task Force (IVETF, 2015), epilepsy in veterinary medicine is divided into the following groups: idiopathic epilepsy (IdE), structural epilepsy (StE), and epilepsy of unknown cause [12]. An epileptic seizure can be defined as visible focal or generalized convulsions of the body’s muscles [12]. Based on seizure frequency and duration, the manifestation can be classified into single generalized (SG), cluster seizures (CS), and status epilepticus (SE). Two or more focal or generalized epileptic seizures within 24 h are defined as CS. SE is defined as seizure activity lasting more than 5 min or as the occurrence of two or more seizures without complete recovery between episodes [5,12,13].

Both CS and SE are associated with poor outcomes and require urgent care. Despite variations in reported outcomes between the CS and SE groups, timely and thorough diagnostic evaluation remains essential for identifying the underlying cause and initiating appropriate treatment [14,15,16].

The latest research on idiopathic epilepsy aims to identify specific genes that would allow for an easier, quicker, and more precise diagnosis [17,18]. A new trend is also observed in the field of nutrition, where compounds with potential benefits in managing epileptic seizures are being investigated [19,20]. Idiopathic epilepsy with single seizure events is usually well-controlled with anti-seizure medications (ASMs) treatment, and treatment guidelines have remained relatively stable over the last decade [21,22]. In many epilepsy studies, good seizure control is defined as a reduction in seizure frequency of more than 50% [23,24,25,26,27]. However, approximately 25–30% of dogs with epilepsy are refractory and do not respond to ASMs [28]. Numerous recent studies have explored alternative drug treatment variations [29,30,31]. Some have suggested that ASMs are not the only viable option; cannabidiol has shown promise in reducing seizure frequency [32,33,34]. Other emerging therapies, such as cyclooxygenase-2 inhibitors, may also positively affect seizure control according to preliminary data [35]. Overall, it has been reported that 6–85% of dogs with epilepsy achieve successful seizure control [21,36].

However, there is evidence that SE and CS are neurological emergencies and may be linked to poor outcomes [15]. Several studies involving SE and CS report high euthanasia rates, particularly in dogs with structural epilepsy [15,37,38,39]. However, extended epidemiologic studies on SE in dogs are limited; one study reports that only 0.44% of all hospitalized patients were admitted with SE [40]. The population-level prevalence of specific seizure types in dogs remains insufficiently characterized; however, estimates vary between studies, with cluster seizures reported in up to 41% of dogs with idiopathic epilepsy and in 66–74.5% of dogs presenting with either CS or SE [37,38,39].

IdE and StE may present differently. It is thought that SE is more commonly seen in the StE group [16]. Among SE cases, generalized seizures were observed more frequently than focal seizures—94% versus 6%, respectively [15]. Many factors such as age, breed, sex, and neuter status have been investigated, and some trends have been noted.

Based on the hypothesis that scientific sources provide quite different information about the types of epileptic seizures diagnosed in dogs, we conducted a retrospective analysis of 254 cases of epilepsy in dogs taken from the Referral Animal Clinic Aisti Vantaa (Finland) and Dr. L. Kriaučeliūnas’ Small Animal Clinic at the Lithuanian University of Health Sciences (Lithuania) in order to assess the type and frequency of epileptic seizures in dogs, taking into account the animal age of seizure onset, sex, weight, neuter status, blood test results, clinical and neurological examinations, magnetic resonance imaging (MRI) findings, etiology classification and clinical outcome.

## 2. Materials and Methods

This retrospective epidemiological study was conducted between 2019 and 2022 using clinical data from two veterinary clinics: the Referral Animal Clinic Aisti (Vantaa, Finland) and the Dr. L. Kriaučeliūnas Small Animal Clinic at the Lithuanian University of Health Sciences (Kaunas, Lithuania).

A structured data collection plan was developed prior to the study. Medical records were retrieved through the digital hospital management systems used in both clinics. The search was performed using predefined keywords related to epileptic seizures in dogs: “epileptic seizures”, “epilepsy”, “status epilepticus”, “cluster seizures”, “episodes”, “neurological seizures”, “loss of consciousness”, “convulsions”, and “involuntary/unconscious seizures”.

Following the initial search, cases were manually reviewed to confirm eligibility based on predefined inclusion criteria. To be included in the study, each case was required to have complete traceable patient information: a unique patient identification code within the hospital system, sex and neuter status (intact or neutered), breed, date of birth, date of clinical visit (used to calculate age at the time of evaluation), body weight, and a detailed medical history. The history had to clearly describe the seizure episodes, including onset, frequency, and progression, enabling accurate classification of seizure type. Seizure onset characteristics were evaluated, including whether the case represented a first-time presentation (not previously treated) or a previously treated case, the latter indicating dogs that had already received anti-seizure medications prior to presentation.

Exclusion criteria were applied in cases where essential demographic data were missing, including age, weight, breed, sex, or neuter status. Additionally, cases were excluded if the nature of the episodes could not be clearly identified or was poorly documented. For example, if seizure-like events—based on the owner’s description or video footage—were determined to be non-epileptic in origin (e.g., paroxysmal dyskinesia, narcolepsy, cataplexy, syncope, or other differential diagnoses), they were not included in the analysis. Furthermore, if the anamnesis recorded by the attending veterinarian lacked sufficient detail to determine the onset and characteristics of the seizure episodes, or if it was not possible to reliably classify the type of seizures at onset, such cases were also excluded.

Only cases with a clear and unquestionable classification of seizure type and onset were retained for final analysis.

Only cases with sufficiently detailed clinical documentation to confidently assign the dog to one of the seizure type categories: single generalized seizures (SG), cluster seizures (CS), or status epilepticus (SE)—were included in the final analysis. Classification of seizure types was based on definitions provided by the International Veterinary Epilepsy Task Force (IVETF) [12].

Within and between the seizure type groups (SG, CS, SE), multiple statistical comparisons were performed to identify significant associations. These analyses included evaluations of demographic characteristics (sex, age, neuter status, body weight), seizure onset patterns (e.g., initial seizure type and progression), clinical examination findings (normal vs. abnormal), blood test results (normal vs. abnormal), neurological examination findings (normal vs. abnormal), MRI status (performed vs. not performed), and MRI outcomes (presence or absence of structural changes in the brain).

In cases where MRI was not performed, patients were classified into the unknown diagnosis category, even if their treatment outcome was known.

Seizure etiology was further classified using the VITAMIN D acronym, which allowed allocation of each case to one of the following diagnostic categories: V—vascular, I—inflammatory, T—trauma/toxic, A—anomaly, M—metabolic, I—idiopathic, N—neoplastic, and D—degenerative [41].

In addition to the above, further analyses were conducted to assess age distribution across the VITAMIN D diagnostic categories; statistical associations between euthanasia rates and epilepsy type (idiopathic vs. structural epilepsy); and age distribution among dogs within the idiopathic and structural epilepsy groups. Furthermore, patients were stratified into two body weight categories—less than or equal to 25 kg and greater than 25 kg—in order to evaluate the potential association between seizure type and body weight [42].

Ethical approval for this study was granted by the Veterinary Section of the Bioethics Centre at the Lithuanian University of Health Sciences (No. 2024-BEC3-T-001).

Data collection and statistical analysis were conducted using Microsoft^®^ Excel 2019 and IBM SPSS Statistics^®^ Edition 29 (2022). Descriptive statistics, including frequency, mean, median, and standard deviation (SD), were calculated where appropriate. Data normality was assessed using the Kolmogorov-Smirnov test. Most variables followed a normal distribution; therefore, mean ± SD was used for data presentation. In cases where normality was not met or for non-parametric data, results were expressed as median with range and interquartile values (Q1 and Q3).

Inferential statistical analysis was performed using the Chi-square test and the Mann-Whitney U test, as appropriate for categorical and non-parametric comparisons. A 95% confidence interval (CI) was applied, and results were considered statistically significant when *p* < 0.05.

Generative artificial intelligence was used to assist in the generation of English-language text, logical structuring of manuscript sections, and the formatting of figures and tables. All scientific content, data, interpretations, and conclusions were provided entirely by the authors. AI (ChatGPT, GPT-5, OpenAI, San Francisco, CA, USA)was used solely to help articulate and visualize the information in a clear and coherent manner. The final manuscript was reviewed, verified, and approved by the authors to ensure scientific accuracy and integrity.

## 3. Results

### 3.1. Seizure Type Distribution

A total of 254 dogs met the inclusion criteria and were included in the study. The cases were distributed between two clinics: 179 (70.5%) from the Finnish Neurology Clinic Aisti (FIN) and 75 (29.5%) from the Lithuanian University of Health Sciences Small Animal Clinic (LTU). The number of subjects from the FIN group was statistically higher than from the LTU group (*p* < 0.001).

Subjects were categorized into three seizure type groups: single generalized seizures (SG), cluster seizures (CS), and status epilepticus (SE). The most frequent seizure type was CS, accounting for 47.2% (*n* = 120) of all cases, followed by SG at 26.8% (*n* = 68) and SE at 26.0% (*n* = 66).

The distribution of seizure types across the two clinics is summarized in Table 1. The Chi-square test did not reveal statistically significant differences between the clinics in terms of seizure type distribution (*p* = 0.180), indicating that the groups are comparable.

### 3.2. Sex and Reproductive Status

Of the 254 dogs included in the study, 55.5% (*n* = 141) were male and 44.5% (*n* = 113) were female. In the FIN group, 54.2% (*n* = 97) were male and 45.8% (*n* = 82) were female. In the LTU group, 58.7% (*n* = 44) were male and 41.3% (*n* = 31) were female. The sex distribution between the two clinics did not differ significantly (*p* = 0.580), indicating that the groups are comparable.

Regarding reproductive status, 69.5% (*n* = 98) of all males were sexually intact (SI), while 30.5% (*n* = 43) were neutered (NEU). Among females, 63.7% (*n* = 72) were sexually intact and 36.3% (*n* = 41) were spayed (SPD). The overall distribution of reproductive status in the total population was 66.9% (*n* = 170) sexually intact and 33.1% (*n* = 84) neutered or spayed.

When analyzed by clinics, a statistically significant difference in reproductive status was observed. In the LTU group, a higher proportion of animals were sexually intact (81.3%, *n* = 61) compared to the FIN group (60.9%, *n* = 109; *p* = 0.002). Specifically, the proportion of spayed females in LTU was significantly lower at 16.1% (*n* = 5) compared to 43.9% (*n* = 36) in FIN (*p* = 0.005). The neutered/spayed rate overall was significantly higher in the FIN group (39.1%) than in LTU (18.7%), these results are shown in Table 2.

When comparing sex distribution within seizure types, no statistically significant differences were observed. In the SG group, 55.9% (*n* = 38) were male and 44.1% (*n* = 30) were female; in the CS group, 57.5% (*n* = 69) were male and 42.5% (*n* = 51) were female; in the SE group, 51.5% (*n* = 34) were male and 48.5% (*n* = 32) were female (*p* = 0.735), Table 3.

Analysis of reproductive status within seizure types also showed no statistically significant differences. In the SG group, 60.3% (*n* = 41) of dogs were sexually intact and 39.7% (*n* = 27) were neutered or spayed. In the CS group, 66.7% (*n* = 80) were intact and 33.3% (*n* = 40) neutered/spayed, and in the SE group, 74.2% (*n* = 49) were intact and 25.8% (*n* = 17) neutered/spayed.

### 3.3. Age Distribution

Age was determined based on the time of presentation to the clinic, when a diagnostic and/or treatment plan was initiated or adjusted.

The Kolmogorov–Smirnov test showed that the overall age distribution was non-normal. Subgroup analysis revealed that the distribution in the FIN cohort was non-normal, while the LTU cohort showed normal distribution. In the LTU group, the mean age was 76.33 months (SD = 48.60), and in the FIN group, the median age was 74 months (Q1 = 37.00; Q3 = 114.00). The overall median age of all subjects was 72.5 months (Q1 = 36.00; Q3 = 114.00). A Mann–Whitney U test showed no statistically significant difference in age distribution between groups (*p* = 0.649), indicating that the groups are comparable.

For further analysis, subjects were categorized into three age groups: ≤6 months, 6–72 months, and >72 months. Distribution was as follows: 3.5% (*n* = 9) were ≤6 months, 46.5% (*n* = 118) were 6–72 months, and 50.0% (*n* = 127) were >72 months. There were no significant differences in age group distribution between clinics (Table 4).

Analysis of seizure type across age groups revealed that SG seizures were significantly more frequent than CS in the 6–72 months age group. As shown in Table 5, SG occurred in 54.4% and CS in 38.3% of this age group (*p* = 0.005).

Diagnosis-based categorization revealed that 46.1% (*n* = 117) of cases were idiopathic epilepsy, 31.9% (*n* = 81) structural epilepsy, and 22.0% (*n* = 56) had unknown etiology.

**Figure 1:**
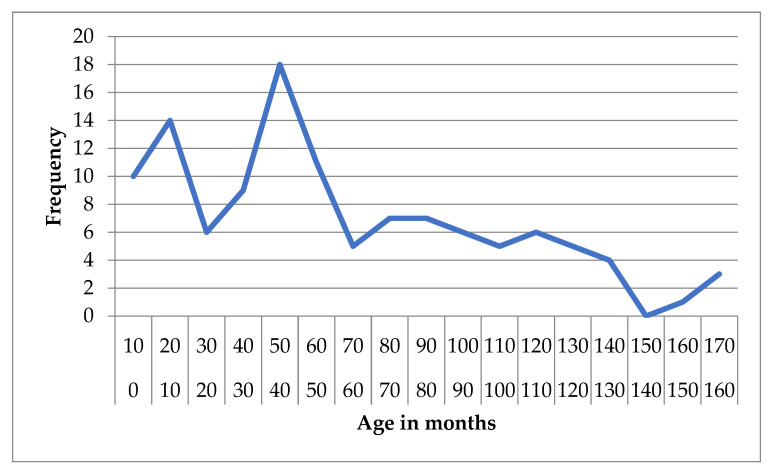
Frequency distribution of age (months) in dogs diagnosed with idiopathic epilepsy.

**Figure 2:**
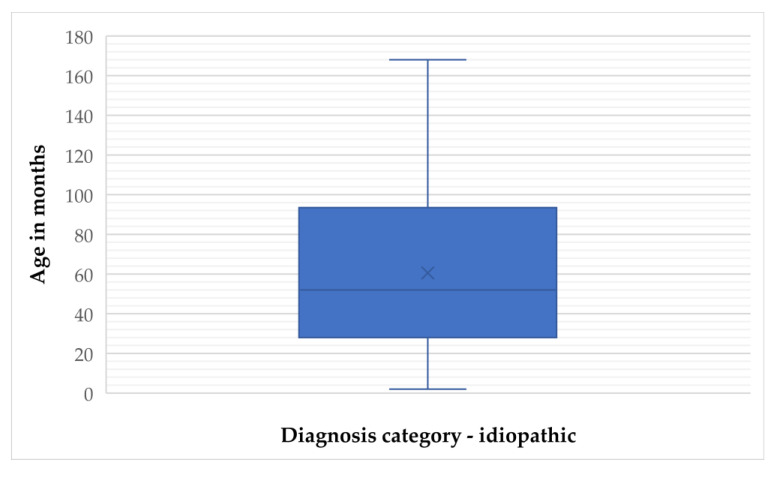
Frequency distribution of age (months) in idiopathic epilepsy cases.

In the idiopathic epilepsy group, the mean age was 60.56 months (SD = 41.92), with a median of 52 months (IQR = 66). Age ranged from 2 to 168 months, and the 95% confidence interval (CI) for the mean was 52.88 to 68.23 months. The 5% trimmed mean was 58.51 months, indicating limited impact of outliers (Figure 1 and Figure 2).

**Figure 3:**
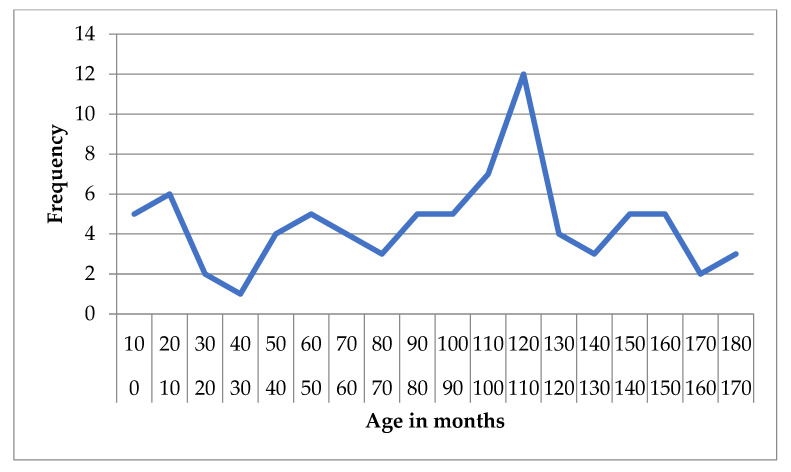
Frequency distribution of age (months) in dogs diagnosed with structural epilepsy.

**Figure 4:**
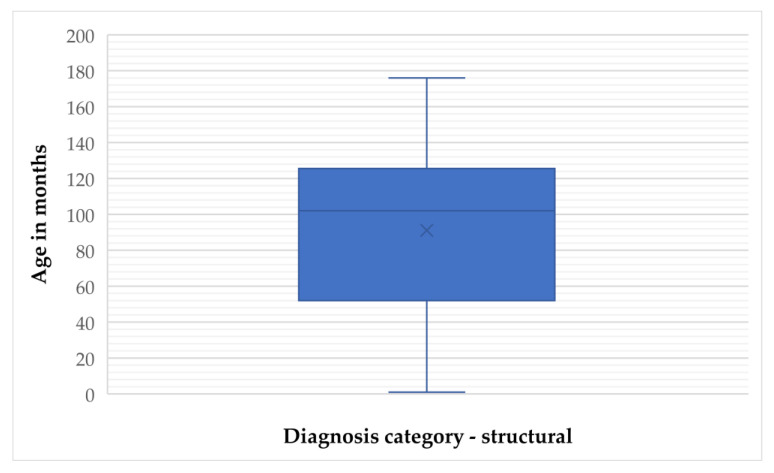
Frequency distribution of age (months) in structural epilepsy cases.

For structural epilepsy, the mean age was 91.06 months (SD = 48.10) and the median was 102 months (IQR = 74), with values ranging from 1 to 176 months. The 95% CI for the mean was 80.43 to 101.70 months, and the 5% trimmed mean was 91.38 months (Figure 3 and Figure 4).

### 3.4. Weight

Body weight data were available for all subjects. The overall median weight was 10.95 kg, with a range from 1.70 to 61.50 kg.

When evaluating seizure types by weight, the median body weight for each group was as follows: SG group—16.45 kg (Q1 = 7.05; Q3 = 25.45), CS group—12.10 kg (Q1 = 8.40; Q3 = 20.00), and SE group—9.85 kg (Q1 = 4.10; Q3 = 19.80). Although a decreasing trend in median body weight was observed with increasing seizure severity, the differences between seizure groups were not statistically significant.

To further assess potential trends, subjects were divided into two weight categories: <25 kg and ≥25 kg. Across all seizure types, there was a tendency for a higher proportion of dogs weighing ≥ 25 kg; however, Chi-square analysis revealed no statistically significant differences between weight categories and seizure types (*p* = 0.337) (Table 6).

### 3.5. Seizure Onset and Clinical Presentation

Seizure onset characteristics were evaluated, including whether the case represented a first-time presentation or prior treatment, initial seizure type, and results of clinical and neurological examination, and blood examinations.

Out of the 254 enrolled subjects, 83.1% (*n* = 211) were first-time (primary) cases and 16.9% (*n* = 43) had been previously treated. Among the primary cases, 30.8% (*n* = 65) presented with single generalized seizures (SG), 44.5% (*n* = 94) with cluster seizures (CS), and 24.6% (*n* = 52) with status epilepticus (SE). Among previously treated cases, SG accounted for only 7.0% (*n* = 3), while CS and SE were more common, at 60.5% (*n* = 26) and 32.6% (*n* = 14), respectively. SG was significantly more prevalent in first-time cases compared to previously treated ones (*p* < 0.005).

Initial seizure type was also assessed: 3.6% (*n* = 9) of cases began with focal seizures, 58.1% (*n* = 148) with SG, 32.4% (*n* = 82) with CS, and 5.9% (*n* = 15) with SE. The evolution of seizure type is summarized in Table 7. Among focal seizure cases, 44.4% (*n* = 4) progressed to SG and 55.6% (*n* = 5) to CS. Of the SG group, 43.5% (*n* = 65) remained SG, 36.7% (*n* = 54) progressed to CS, and 19.7% (*n* = 29) progressed to SE. Among CS cases, 73.2% (*n* = 60) remained CS and 26.8% (*n* = 22) progressed to SE. All cases initially presenting as SE remained within the SE group. Seizure progression from CS to SE was statistically significant (*p* < 0.05).

#### 3.5.1. Blood Test Findings

Subjects were categorized into two blood test groups: normal and abnormal results. No statistically significant differences were found in blood test status between seizure groups overall. However, within the abnormal group, SE cases accounted for 62.5% of the findings, while CS and SG accounted for 31.3% and 6.3%, respectively (*p* < 0.05).

#### 3.5.2. Clinical Examination Findings

Only 7.1% (*n* = 18) of subjects exhibited abnormal clinical findings; the majority (92.9%, *n* = 236) had normal clinical examinations. Abnormal clinical findings were significantly more frequent in the SE group (55.6%, *n* = 10) compared to the SG (22.2%, *n* = 4) and CS (22.2%, *n* = 4) groups (*p* < 0.05).

#### 3.5.3. Neurological Examination Findings

Neurological examination revealed abnormalities in 59.1% (*n* = 150) of subjects and normal results in 40.9% (*n* = 104). Abnormal neurological findings were most common in the SE group (83.3%), compared to 53.3% in the CS group and 45.6% in the SG group. These differences were statistically significant (*p* < 0.05).

### 3.6. Diagnostics and Etiological Classification

**Figure 5:**
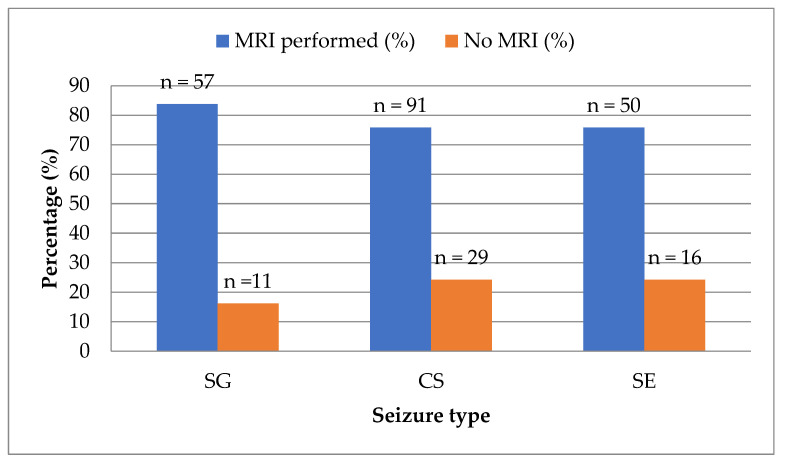
MRI performance across seizure type groups (SG—single generalized seizure, CS—cluster seizures, SE—status epilepticus).

Magnetic resonance imaging (MRI) was performed in 198 of 254 subjects (78.0%), while 56 dogs (22.0%) did not undergo MRI. There was no statistically significant difference in MRI performance between seizure type groups (Figure 5).

Among the cases that underwent MRI (*n* = 198), 53.5% (*n* = 106) had no visible brain lesions, and 46.5% (*n* = 92) exhibited structural abnormalities. Although no statistically significant association was found between seizure type and the presence of MRI abnormalities, a trend was noted: more complex seizure types (CS and SE) were more frequently associated with MRI-detectable brain changes (Table 8).

Based on clinical assessment and available diagnostic data, cases were assigned to etiological categories using the VITAMIN D framework. The distribution of diagnosis categories among all cases (*N* = 254) is summarized in Table 9. Idiopathic epilepsy was the most common diagnosis (45.7%, *n* = 116), followed by unknown etiology (22.1%, *n* = 56), neoplastic (16.1%, *n* = 41), and inflammatory conditions (11.4%, *n* = 29). Vascular, trauma/toxic, anomaly, and metabolic causes were rare, and no cases were classified as degenerative.

**Figure 6:**
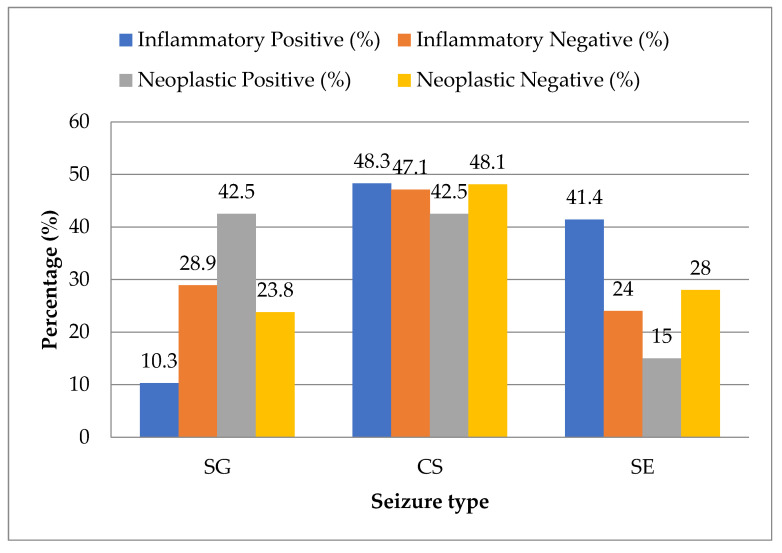
Frequency distribution of seizure types in relation to inflammatory and neoplastic diagnosis groups.

Statistical analysis showed that idiopathic epilepsy was evenly distributed across all seizure types with no significant association. However, in the inflammatory category, SE was significantly more prevalent (*p* < 0.05), while SG was underrepresented (*p* < 0.05). In contrast, neoplastic epilepsy was significantly more likely to present with SG and significantly less likely with SE (*p* < 0.05). No statistically significant trend was found in CS for either group (Figure 6).

### 3.7. Outcome

Treatment outcomes were known for 62.2% (*n* = 158) of the total 254 subjects, while outcomes remained unknown for 37.8% (*n* = 96). Among the known outcomes, 52.5% (*n* = 83) were classified as controlled cases, 13.9% (*n* = 22) required major treatment adjustments, 29.2% (*n* = 46) resulted in euthanasia, and 4.4% (*n* = 7) died. Statistical analysis revealed a significant association between seizure type and euthanasia outcome, with euthanasia being most frequent in the status epilepticus (SE) group (*p* < 0.05).

Of the euthanized subjects (*n* = 46), 54.3% (*n* = 25) were euthanized due to poor prognosis, while 45.7% (*n* = 21) were due to uncontrolled seizure activity. Among patients with cluster seizures (CS), poor prognosis was the leading cause of euthanasia (63.2%), whereas in SE patients, the dominant cause was seizure refractoriness (52.4%). In the single generalized seizure (SG) group, both causes were equally represented (50% each). These findings suggest that the clinical presentation and complexity of seizure types played a role in the decision-making process regarding euthanasia.

Further analysis of euthanized dogs diagnosed with idiopathic epilepsy (IdE) demonstrated a broad age range. The mean age was 53 months, with a median age of 40 months, indicating that more than half of these patients were younger than 3.5 years. Age ranged from 12 to 129 months. The slight difference between the mean and median suggests a moderate skew toward older individuals.

In contrast, euthanized patients with structural epilepsy (StE) had a higher mean age of 94 months and a median of 108 months. The age range was from 1 to 162 months, indicating that structural epilepsy was more frequently diagnosed in older dogs and that euthanasia decisions were commonly made in senior individuals. The negative skewness of the age distribution (median > mean) further supports this observation, showing a predominance of older patients.

## 4. Discussion

Effect of sex on seizures

The current literature suggests an over-representation of male dogs with recurrent seizures. In addition, studies on certain breeds have also demonstrated a significantly higher prevalence of seizures in males, including Bernese Mountain Dogs [43], Finnish Spitz [44], Golden Retrievers [45], Irish Wolfhounds [46], and Italian Spinone [47]. Conversely, there are a number of studies which did not find a statistically significant difference in sex representation in their study populations [4,48]. In our study, a higher number of male dogs in comparison with females were included; however, the difference was not statistically significant. In addition, no significant influence of sex on seizure type was not detected, which is consistent with findings from previous studies [49,50].

Effect of neuter status on seizures

Evidence from other studies supports the effect of neuter status on seizure occurrence, especially in female dogs [51], suggesting that sex hormones might contribute to the development of seizures. In addition, Monteiro et al. found that intact males were twice as likely as neutered dogs to suffer from cluster seizures, and intact females had significantly more frequent cluster seizures compared to neutered dogs [37]. However, our study did not identify such an association. However, our findings are consistent with those studies that have reported that neuter status is not a significant factor influencing seizure occurrence or seizure type [11,39,52]. Nevertheless, it was not possible to determine whether the dogs in our study were neutered before or after experiencing their first seizure, making it difficult to draw conclusions regarding neuter status as a contributing factor to seizure development.

Body weight

Unexpectedly, body weight has been identified in the literature as a potential risk factor contributing to seizure severity. Saito et al. reported that dogs with higher body weights were more likely to experience episodes of status epilepticus (SE) [42]. Similarly, Erlen et al. found that dogs weighing over 40 kg were at higher risk for seizures compared to those under 10 kg [53]. The results of our study are also consistent with previous reports, indicating an increased incidence of seizure in dogs weighing more than 25 kg. No clear association between body weight and seizure type was observed, because statistical significance was not established. The underlying reason why larger dogs may be more prone to seizures remains unclear, though this trend may be partially explained by the overrepresentation of large breeds predisposed to idiopathic epilepsy.

Age onset

Age onset is often associated with the type of epilepsy. Idiopathic epilepsy is generally considered to occur between 6 months and 6 years of age, whereas seizure onset outside this range—particularly in older dogs—may suggest an increased likelihood of structural epilepsy. In our study, more than half of the whole study population were dogs outside the “idiopathic age.” Dogs over 72 months (6 years) consisted of 50.0% of all cases, and dogs up to 6 months of age consisted of 3.5%. Our results are in accordance with a publication by Cagnotti et al. [15], where a similar distribution of cases between age groups has been shown. The median age in this study was 72.5 months (6 years), while the median age for idiopathic epilepsy dogs was 52 months (4.3 years). There is a substantial discrepancy compared to other studies where the median age typically ranges from 2.5 to 3.7 years depending on breed [54,55]. This notable difference in our study may be attributed to the inclusion of dogs with structural epilepsy, which tends to occur later in life. Additionally, the higher median age observed in our idiopathic epilepsy group may be influenced by the inclusion of patients already undergoing treatment for seizures (16.9% (*n* = 43) were previously treated cases).

Initial type of seizure in whole study population

The nature and severity of seizures at the onset of the disease course can vary greatly and are influenced by a range of, often unknown, factors. In our study, only 3.6% (*n* = 9) of all cases initially started as focal seizures. The majority of dogs in the population of this study (58.1%, *n* = 148) experienced generalized seizures as their initial seizure type. This finding is consistent with results from other large population-based and breed-specific studies [56,57,58,59,60]. The predominance of generalized seizures may reflect their higher likelihood of being recognized by dog owners, whereas focal seizures, especially those that secondarily generalize, may be overlooked or underdiagnosed during the early course of the disease. This is particularly true when focal seizures occur at night or present with non-specific autonomic or behavioral signs such as anxiety, restlessness, unexplained fear reactions, dilated pupils, vomiting, or other subtle manifestations [12].

In our dataset, cluster seizures (CS) were the most frequently observed seizure type (47.2%), followed by single generalized (SG) seizures (26.8%) and status epilepticus (SE) (26.0%). As the data were collected exclusively from two referral veterinary clinics, it is possible that the relative frequency of CS and SE could be higher compared to the general canine population. Referral institutions typically manage patients with more severe or refractory epilepsy, which may explain the predominance of CS and SE in our study cohort. This should be taken into consideration when interpreting the generalizability of our findings.

Initial type of seizure compared between primary and previously treated groups

Conversely, when the initial seizure type was compared between the primary and previously treated groups, a tendency was observed—though not statistically significant—for seizures to initially present as cluster seizures rather than as focal or generalized seizures in the primary seizure group (generalized seizures: 30.8% [*n* = 65], cluster seizures: 44.5% [*n* = 94], status epilepticus: 24.6% [*n* = 52]). This trend is supported by a previous study on Australian Shepherds, which found that 28% of dogs experienced cluster seizures or status epilepticus as their first seizure event; however, no breed distribution was evaluated in this study [61].

Type of seizure at the time of diagnosis compared between primary and previously treated groups. When seizure type at the time of diagnosis was compared between primary and previously treated groups, generalized seizures were significantly more common in the primary group. However, when assessing the total study population, generalized seizures were not the most frequently observed type overall. This apparent discrepancy may indicate that seizure presentation evolves over time, with generalized seizures occurring more frequently at the onset of the disease, and more severe forms, such as cluster seizures or status epilepticus becoming increasingly prevalent as the disease progresses. Supporting this, in the previously treated group, cluster seizures occurred in 60.5% (*n* = 26) and status epilepticus in 32.6% (*n* = 14), together accounting for the majority of cases. Additionally, in our study, cluster seizures were significantly more likely to progress to status epilepticus over the course of the disease (*p* < 0.05).

Type of seizure and age

Conversely, when we looked at the most common seizure types between age groups, there was a significantly greater number of generalized seizures compared with cluster seizures in dogs up to 72 months (6 years) of age. This may suggest that younger dogs are more likely to initially present with generalized seizure activity rather than with more severe or repetitive seizure manifestations such as cluster seizures. However, seizure type may vary between breeds, as genetic and breed-specific factors can influence seizure presentation and progression. Although the difference between age groups and seizure type was not statistically significant. Our findings may reflect a tendency for more severe seizure types, such as CS and SE, to develop over time, possibly due to disease progression or delayed recognition and suboptimal management. In accordance with this observation, dogs over 72 months of age exhibited a slightly higher frequency of CS and SE compared to GS in our study. However, conversely, results of the other researchers indicate that dogs with a history of CS were younger at onset than those with other types of seizure [39]. This discrepancy may also be influenced by breed-related factors, underscoring the complexity and variability of seizure expression across canine populations.

Blood test findings

Routine laboratory blood tests, along with other basic diagnostic evaluations, are generally recommended as a minimum database for nearly all clinical presentations, including patients with seizures. Hematologic or biochemical abnormalities may suggest a metabolic cause of seizures; however, to date, there is no consistent complete blood count (CBC) or serum biochemistry profile characteristic of dogs with seizures. In fact, most dogs with seizures show no notable changes on routine bloodwork. Interestingly, a recent study by Despa et al. [62] reported that the neutrophil-to-lymphocyte ratio was elevated in all epileptic patients, regardless of etiology or clinical presentation. In contrast, C-reactive protein concentrations were found to be abnormal only in dogs presenting with cluster seizures.

Consistent with previous findings, our study did not identify any specific biomarkers in routine blood examinations that could distinguish dogs with seizures. Abnormal blood test results were significantly more common in dogs with status epilepticus compared to those with generalized or cluster seizures (*p* < 0.05), suggesting that systemic alterations may be more pronounced in cases of prolonged or severe seizure activity.

In addition, a similar pattern was observed in both clinical and neurological examinations: dogs with status epilepticus were significantly more likely to exhibit abnormalities on physical and neurological assessments (*p* < 0.05)

Differential diagnosis and seizure types

Seizures may result from a variety of underlying causes, which is why advanced imaging, particularly MRI, is essential to determine the actual cause. In our study, 53.5% (*n* = 106) of dogs had normal MRI scans, while 46.5% (*n* = 92) showed brain abnormalities. Using the VITAMIN D [41] framework to categorize MRI findings based on lesion type, onset, and progression of symptoms allowed us to assign them to specific differential diagnosis groups. We observed significant associations between seizure type and diagnosis category: dogs with inflammatory brain disease were more likely to present with status epilepticus and less likely to have generalized seizures (*p* < 0.05), whereas dogs with neoplastic disease were significantly more likely to present with generalized seizures instead of status epilepticus (*p* < 0.05). This may be explained by the rapid progression of inflammatory processes, which can impair compensatory mechanisms and lower the seizure threshold, thereby facilitating the development of severe seizure activity, whereas neoplastic growth is often slower, potentially allowing for adaptive responses that initially might manifest as less severe seizure types [63,64]. Because most existing research considers inflammatory and neoplastic conditions collectively within the structural epilepsy category, direct comparisons with our results are limited. This methodological difference may explain why the distinct associations observed in our study have not been highlighted in earlier publications.

In line with the findings presented by Armaşu et al. [65], we observed that cluster seizures were more prevalent than single generalized seizures among dogs with structural etiologies, especially in older age groups. This suggests consistent epidemiological patterns across different cohorts of epileptic dogs.

Outcome

Of all known outcomes in our study, euthanasia accounted for 29.2% (*n* = 46) of cases and was significantly more common among dogs that experienced status epilepticus (*p* < 0.05). These findings are consistent with previous publications, which have reported status epilepticus as a factor associated with increased short-term mortality [50,51,66]. Fentem et al. also noted that increasing age at the time of SE was a significant risk factor influencing short-term survival [66]. Similarly, Cagnotti et al. [15] found that age at onset, outside the idiopathic interval was linked to poor prognosis. In our study, although the age of euthanized dogs varied between the idiopathic and structural epilepsy groups, a tendency was detected for euthanized dogs within the structural epilepsy group to be older than those in the idiopathic group, although, the difference was not statistically significant.

In our study the treatment outcomes remained unknown for the 37.8% (*n* = 96) of patients. All known treatment outcomes (62.2% (*n* = 158)) in our study, euthanasia accounted for 29.2% of cases (*n* = 46) and was significantly more common among dogs that experienced status epilepticus (*p* < 0.05). These findings are consistent with previous publications, which have reported status epilepticus as a factor associated with increased short-term mortality [50,51,66].

Fentem et al. also noted that increasing age at the time of SE was a significant risk factor influencing short-term survival [66]. Similarly, Cagnotti et al. [15] found that age at onset, outside the idiopathic interval was linked to poor prognosis.

In our study, although the age of euthanized dogs varied between the idiopathic and structural epilepsy groups, a tendency was detected for euthanized dogs within the structural epilepsy group to be older than those in the idiopathic group, although, the difference was not statistically significant.

Nevertheless, a large proportion (62.2%) of cases with known outcomes and the results of other previous studies [50,51,66] suggests the reliability of our conclusions, especially the higher euthanasia rate observed in dogs with SE and structural epilepsy.

## 5. Conclusions

This study demonstrated that cluster seizures were the most common seizure presentation in the investigated dog population, whereas status epilepticus was strongly associated with inflammatory brain disease, abnormal neurological findings, and a higher risk of euthanasia. Dogs with structural epilepsy were generally older and more likely to develop severe seizure forms. Although no significant associations were observed between seizure type and sex, neuter status, or body weight, the high proportion of dogs outside the typical idiopathic epilepsy onset age highlights the importance of thorough diagnostic evaluation in all age groups. Progression from single generalized seizures to cluster seizures or status epilepticus was common, underscoring the dynamic nature of seizure manifestation. These findings emphasize the need for early recognition, comprehensive diagnostics—including advanced imaging—and individualized treatment strategies to improve outcomes in canine epilepsy.

## Figures and Tables

**Table 1:** Seizure type distribution across FIN and LTU groups (SG—single generalized seizures, CS—cluster seizures, SE—status epilepticus).

Seizure Type	FIN (*n* = 179)	LTU (*n* = 75)	Total (*n* = 254)	χ^2^/*p*
SG	46 (25.7%)	22 (29.3%)	68 (26.8%)	χ^2^ = 3.400
CS	91 (50.8%)	29 (38.7%)	120 (47.2%)	*p* = 0.180
SE	42 (23.5%)	24 (32.0%)	66 (26.0%)	

**Table 2:** Distribution of reproductive status between study sites (SI—sexually intact; NEU—neutered; SPD—spayed).

	FIN (*n* = 179)	LTU (*n* = 75)	χ^2^/*p*
Males			
SI	63 (64.9%)	35 (79.5%)	χ^2^ = 3.043
NEU	34 (35.1%)	9 (20.5%)	*p* = 0.081
Females			
SI	46 (56.1%)	26 (83.9%)	χ^2^ = 7.506
SPD	36 (43.9%)	5 (16.1%)	*p* = 0.005
Total			
SI	109 (60.9%)	61 (81.3%)	χ^2^ = 9.976
NEU/SPD	70 (39.1%)	14 (18.7%)	*p* = 0.002

**Table 3:** Distribution of sex across seizure type groups.

Seizure Type	Male (*n* = 141)	Female (*n* = 113)	Total (*n* = 254)	χ^2^/*p*
SG	38 (27.0%)	30 (26.5%)	68 (26.8%)	χ^2^ = 3.400
CS	69 (48.9%)	51 (45.1%)	120 (47.2%)	*p* = 0.735
SE	34 (24.1%)	32 (28.3%)	66 (26.0%)	

**Table 4:** Distribution of subjects by age group and institution.

Age Group	FIN (*n* = 179)	LTU (*n* = 75)	χ^2^/*p*
≤6 months	8 (4.5%)	1 (1.3%)	χ^2^ = 1.96
6–72 months	80 (44.7%)	38 (50.7%)	*p* = 0.376
>72 months	91 (50.8%)	36 (48.0%)	

**Table 5:** Age group distribution across seizure types. * Statistically significant difference.

Age Group	SG	CS	SE	Total (*n*)	χ^2^/*p*
≤6 months	0	9 (7.5%)	0	9	χ^2^ = 14.10
6–72 months	37 (54.4%) *	46 (38.3%) *	35 (53.0%)	118	*p* = 0.005
>72 months	31 (45.6%)	65 (47.2%)	31 (47.0%)	127	

**Table 6:** Distribution of seizure types by body weight group (<25 kg and ≥25 kg).

Weight Group	SG (*n* = 68)	CS (*n* = 120)	SE (*n* = 66)	χ^2^/*p*
≥25 kg	50 (73.5%)	99 (82.5%)	53 (80.3%)	χ^2^ = 2.18
<25 kg	18 (26.5%)	21 (17.5%)	13 (19.7%)	*p* = 0.337

**Table 7:** Seizure progression from initial to subsequent seizure types.

Started as	Progressed to	%
Focal seizures	CS/SG	55.6%/44.4%
Single generalized	Remained SG → CS → SE	43.5% → 36.7% → 19.7%
Cluster seizures	Remained CS → SE	73.2% → 26.8%
Status epilepticus	Remained SE	100.0%

**Table 8:** Distribution of abnormal MRI findings by seizure type.

Seizure Type	Abnormal MRI (*n*)	% of Total Abnormal MRIs
SG	27	29.3%
CS	37	40.2%
SE	28	30.5%

**Table 9:** Distribution of diagnostic categories in all dogs with epilepsy according to the VITAMIN D classification framework.

Diagnosis Category	Number of Cases	Percentage (%)
Idiopathic	116	45.67
Unknown	56	22.05
Neoplastic	41	16.14
Inflammatory	29	11.42
Trauma/Toxic	4	1.57
Anomaly	5	1.97
Metabolic	2	0.79
Vascular	1	0.39
Degenerative	0	0.00

## Data Availability

The data presented in this study are available on request from the corresponding author. The data are not publicly available due to privacy or ethical restrictions.

## Abbreviations

The following abbreviations are used in this manuscript:

ASMs	Anti-seizure medications
CBC	Complete blood count
CI	Confidence Interval
CRP	C-Reactive Protein
CS	Cluster Seizures
IdE	Idiopathic Epilepsy
IE	Idiopathic Epilepsy
IVETF	International Veterinary Epilepsy Task Force
MRI	Magnetic Resonance Imaging
NEU	Neutered
Q1, Q3	First and Third Quartile
SD	Standard Deviation
MCT	Medium-Chain Triglyceride
NLR	Neutrophil-to-Lymphocyte Ratio
SE	Status Epilepticus
SG	Single Generalized (seizure)
SI	Sexually Intact
SPD	Spayed
StE	Structural Epilepsy
VITAMIN D	Vascular, Inflammatory, Trauma/Toxic, Anomaly, Metabolic, Idiopathic, Neoplastic, Degenerative
